# A truncated PPAR gamma 2 localizes to mitochondria and regulates mitochondrial respiration in brown adipocytes

**DOI:** 10.1371/journal.pone.0195007

**Published:** 2018-03-22

**Authors:** Ji Suk Chang, Kyoungsoo Ha

**Affiliations:** Laboratory of Gene Regulation and Metabolism, Pennington Biomedical Research Center, Baton Rouge, Louisiana, United States of America; Universidad Pablo de Olavide, SPAIN

## Abstract

Peroxisome proliferator-activated receptor gamma (PPARγ) is a key regulator of brown adipocyte differentiation and thermogenesis. The PPARγ gene produces two isoforms, PPARγ1 and PPARγ2. PPARγ2 is identical to PPARγ1 except for additional 30 amino acids present in the N-terminus of PPARγ2. Here we report that the C-terminally truncated form of PPARγ2 is predominantly present in the mitochondrial matrix of brown adipocytes and that it binds to the D-loop region of mitochondrial DNA (mtDNA), which contains the promoter for mitochondrial electron transport chain (ETC) genes. Expression of mitochondrially targeted MLS-PPARγ2 in brown adipocytes increases mtDNA-encoded ETC gene expression concomitant with enhanced mitochondrial respiration. These results suggest that direct regulation of mitochondrially encoded ETC gene expression by mitochondrial PPARγ2, in part, underlies the isoform-specific role for PPARγ2 in brown adipocytes.

## Introduction

A growing body of evidence from recent studies reveals that nuclear transcription factors translocate to mitochondria and play a role in a cell- or tissue-specific manner. Thyroid receptor (TRα isoform p43), MEF2D, STAT3, and CREB translocate to mitochondria and directly modulate mitochondrial DNA transcription in response to specific stimuli [1–5], indicating that nuclear transcription factors can control mitochondrial function via transcriptional regulation of mitochondrial genome. In addition, nuclear transcription factors have non-genomic function in mitochondria. Stress-induced p53 has been shown to trigger apoptosis in mitochondria by destabilizing the outer mitochondrial membrane through interaction with multi-domain Bcl-2 family members [6]. Estrogen receptors (ERα and ERβ) can affect mitochondrial fatty acid β-oxidation by directly regulating mitochondrial HADHB enzyme activity [7–9]. Furthermore, a growing number of nuclear receptors such as glucocorticoid receptor (GR), vitamin D receptor (VDR), retinoid X receptor (RXR) and retinoic acid receptor (RAR) have been found in the mitochondria of various cells and tissues, although their function in mitochondria has not been explored [10].

Peroxisome proliferator-activated receptor (PPARγ) is a key regulator of terminal fat cell differentiation [11, 12]. PPARγ also regulates many genes involved in thermogenesis, lipid transport and metabolism, and insulin signaling in brown adipocytes [13, 14]. Alternative promoter usage and alternative splicing give rise to two different PPARγ isoforms, PPARγ1 and PPARγ2. These two isoforms are identical except for additional 30 amino acids present in the N-terminus of PPARγ2 compared to PPARγ1 [15]. PPARγ1 and PPARγ2 are induced during differentiation of brown preadipocytes [12], but functional differences of these two isoforms have not been investigated. A previous study reported that a 45 kDa protein related to PPARγ2 is present in the mitochondria of several rat tissues including brown adipose tissue [16]. In the present study, we found that a C-terminally truncated form of PPARγ2 is predominantly present in the mitochondria of brown adipocytes and thus sought to investigate the possible isoform-specific role for PPARγ2 in brown adipocyte mitochondria.

## Materials and methods

### Mice

All animal experiments were performed according to the procedures reviewed and approved by the Pennington Biomedical Research Center Institutional Animal Care and Use Committee (PBRC IACUC). C57BL/6J mice were housed on a 12-h light/12-h dark cycle. To collect brown adipose tissue from the interscapular region, mice were sacrificed with CO_2_ inhalation, followed by cervical dislocation. The animal study was approved by the PBRC IACUC in the protocol 659 (03/23/2010) and protocol 740 (08/22/2011).

### Cell culture

HeLa cells (ATCC) were grown in DMEM supplemented with 10% FBS and 1% penicillin/streptomycin and transfected using Fugene 6 (Roche Applied Science). Immortalized mouse brown preadipocytes [17] were grown in DMEM supplemented with 10% FBS and 1% penicillin/streptomycin and induced for differentiation as described previously [17, 18].

### Plasmid construction

PPARγ2 was amplified from pCMX-PPARγ2 using primers containing SalI and NotI sites and subcloned into SalI/NotI sites of pCMV/mito that contains the mitochondrial localizing sequence (MLS). For a retroviral plasmid of pBABE-MLS-PPARγ2-HA, MLS-PPARγ2-HA was amplified from pCMV/mito-PPARγ2-HA using primers containing SnaBI and XhoI sites and subcloned into SnaBI/SalI sites of pBABE-neo. All plasmids generated were sequenced to rule out any mutations.

### Subcellular fractionation

To obtain nuclear, cytosolic and mitochondrial fractions, tissues or cells were homogenized and subjected to subcellular fractionation by differential centrifugation as described previously [19].

### Immunofluorescence

Brown adipocytes were seeded on glass coverslips, fixed, and subjected to indirect immunofluorescence as described previously [19]. The cells were analyzed with a Plan-Neofluar 40×/0.85 numerical aperture objective on a Zeiss LSM510 Meta confocal microscope.

### Proteinase K digestion assay

Purified mitochondria were resuspended in SEM buffer (250mM sucrose, 1mM EDTA, 10mM MOPS, protease inhibitors) and incubated with increasing amounts of proteinase K for 15 min on ice in the absence or presence of 1% Triton X-100. After addition of 2mM PMSF, mitochondria were spun down, rinsed with SEM buffer containing PMSF, and resuspended in 2x Laemmli sample buffer.

### Transmission electron microscopy and immunolabeling

Immuno-transmission electron microscopy (TEM) was carried out as described previously [19]. Briefly, brown adipocytes were fixed in 2% glutaraldehyde and 1% paraformaldehyde, followed by 1% osmium tetroxide. After dehydration, the cells were embedded in resin. Thin sections from the resin blocks were mounted on nickel grids. The grids were then subjected to immuno-labeling by incubating with PPARγ (H-100) antibody or rabbit IgG. The grids were analyzed using a JEOL JEM 2011 transmission electron microscope at the LSU Socolofsky Microscopy Center.

### Western blot

Cells were subjected to Western blot analysis as described previously [17]. Antibodies used were as follows: anti-PPARγ2 (PA1-824) from Thermo Fisher Scientific, anti-PPARγ (H-100) (sc-7196), anti-PPARγ (E-8) (sc-7273), anti-Tom20, anti-Lamin B1 from Santa Cruz, anti-UCP1[20], and anti-HSP60 from Abcam.

### Mitochondrial chromatin immunoprecipitation assay

Mitochondrial chromatin immunoprecipitation was carried out as described previously [19]. Briefly, mitochondria were isolated from brown adipocytes, crosslinked with 1% formaldehyde, and sheared to obtain chromatin fragments ranged from 400 bp to 1 kbp. After centrifugation at 10,000 × g, the supernatant was pre-cleared with BSA-blocked Protein A agarose beads and incubated with a ChIP grade anti-PPARγ (H-100) antibody or IgG at 4°C. The crosslinked DNA-protein complexes were released from BSA-blocked Protein A agarose beads and the DNA samples were purified. Quantitative real-time PCR was carried out using a pair of primers specific for the D-loop region and for ND1. D-loop fwd: 5′-gtggtgtcatgcatttggtatct-3′; D-loop rev: 5′-catgaataattagccttaggtgat-3′; ND1 fwd: 5′-cccattcgcgttattctt-3′; ND1 rev: 5′-aagttgatcgtaaggaagc-3′.

### Retroviral infection

Retroviruses expressing an empty vector (pBABE-neo) or pBABE-MLS-PPARγ2-HA were produced as described previously [17]. Immortalized brown preadipocytes were infected in retrovirus-containing medium supplemented with 8 μg/ml of polybrene for 8h as described previously [17]. After 48h, neomycin resistant clones were selected and pooled.

### Quantitative real-time PCR

Total RNA was extracted from cells using the RNeasy mini kit with DNase I treatment (Qiagen). cDNA synthesis and quantitative real-time PCR were carried out as described previously [17, 18, 21]. Relative abundance of mRNA was determined after normalization to that of cyclophilin mRNA using the ΔΔCt method.

### Oxygen consumption assay

Cellular oxygen consumption assays were performed using the OROBOROS Oxygraph-2k (Oroboros Instruments, Innsbruck, Austria) as described previously [22]. Briefly, brown adipocytes (10^6^ cells) were placed in a magnetically stirred respirometric chamber and measured for oxygen consumption rates (OCR) at baseline and after injection of antimycin A (a mitochondrial electron transport inhibitor). The value of mitochondrial respiration was determined by subtracting antimycin A-independent non-mitochondrial respiration as described in the Oroboros Operator’s Manual.

### Statistical analysis

All data are presented as mean ± SEM. Student *t* tests were used to compare the difference between groups using Graphpad Prism 6 software. Values of *P* < 0.05 were considered statistically significant.

## Results

### A C-terminally truncated form of PPARγ2 is predominantly present in brown adipose tissue mitochondria

**Fig 1:**
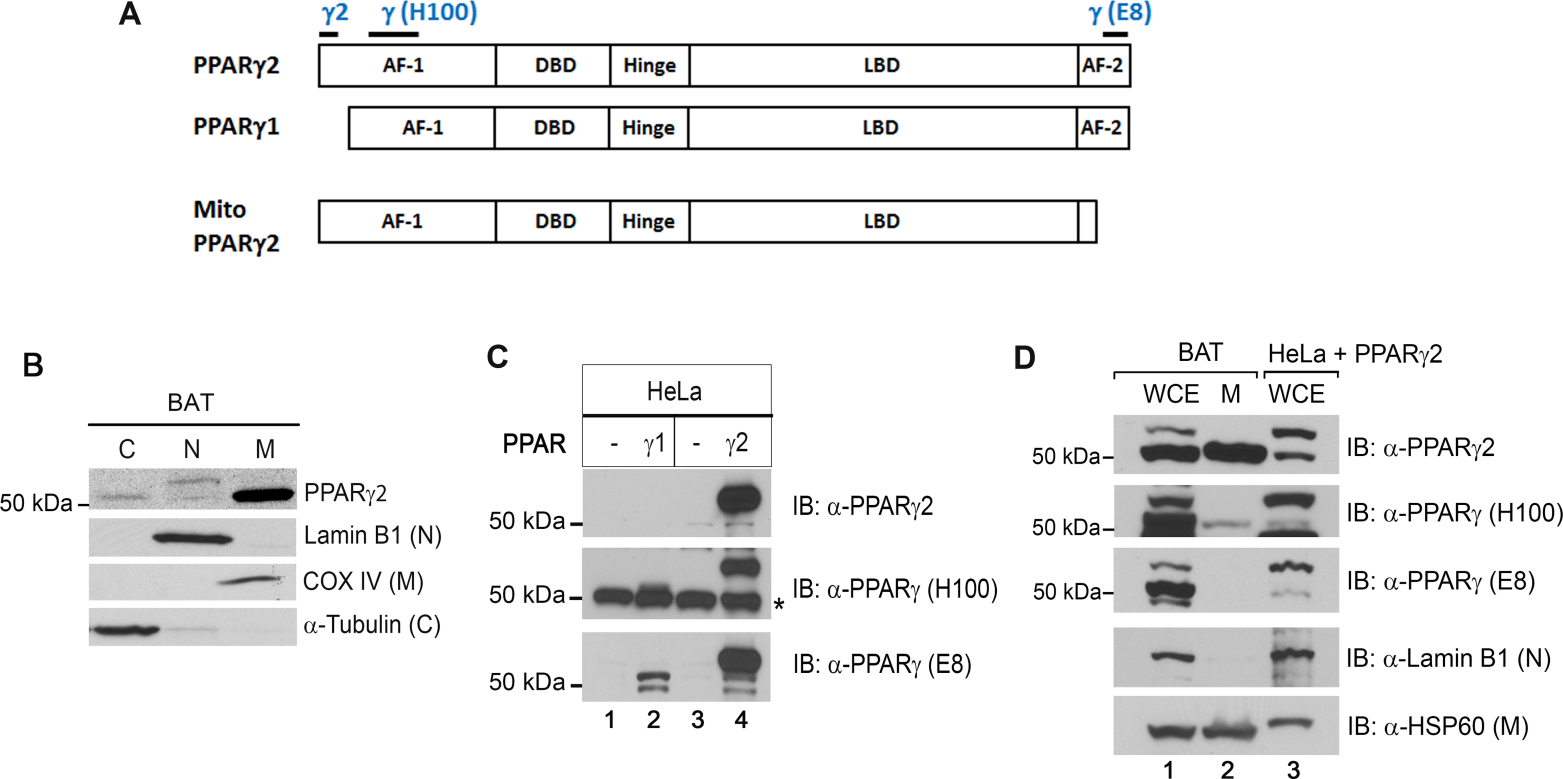
A C-terminally truncated form of PPARγ2 is enriched in brown adipose tissue mitochondria. (A) Schematic of PPARγ1 and PPARγ2 proteins. AF-1, activation function 1; DBD, DNA binding domain; LBD, ligand binding domain; AF-2, activation function 2. Blue letters represent specific regions recognized by three different PPARγ antibodies. (B) Presence of a 52 kDa protein recognized by PPARγ2 antibody in the cytosolic and mitochondrial fractions. Brown adipose tissue was isolated from C57BL/6J mice and subjected to subcellular fractionation. Cytosolic (C), nuclear (N) and mitochondrial (M) markers were detected in their respective fractions. (C) Validation of three different PPARγ antibodies. PPARγ1 and PPARγ2 were expressed in HeLa cells and analyzed with three different PPARγ antibodies. *, a non-specific band at ~50kDa that is reacted with PPARγ (H100) antibody in HeLa cells. (D) Western blot analysis of brown adipose tissue extracts (WCE) and mitochondrial lysates (M) with three different PPARγ antibodies.

The PPARγ gene produces two different isoforms, PPARγ1 and PPARγ2. PPARγ2 has additional 30 amino acids at the N-terminus compared to PPARγ1 (Fig 1A). Subcellular fractionation of brown adipose tissue (BAT) and western blot analysis with anti-PPARγ2 antibody unexpectedly revealed that while a ~57 kDa protein representing PPARγ2 was enriched in the nuclear fraction, a ~52 kDa protein was predominantly present in the cytoplasmic and mitochondrial fractions (Fig 1B). The nuclear marker Lamin B1 was not detected in the cytoplasmic and mitochondrial fractions, indicating no contamination of these fractions by nuclei. To determine if a lower molecular weight band represents a variant of PPARγ2, we first tested the specificity of PPARγ2 antibody that recognizes an epitope corresponding to amino acids 1–16 of PPARγ2 (Fig 1A). PPARγ1 and PPARγ2 were separately expressed in HeLa cells and their protein expression was analyzed using PPARγ2 antibody. PPARγ1was not detected by PPARγ2 antibody, but PPARγ2 was strongly detected at 57 kDa (Fig 1C, top panel), confirming that this antibody is highly specific to PPARγ2. Immunoblotting of BAT whole cell extracts with PPARγ2 antibody showed two bands at 57 and 52 kDa, and the lower molecular weight protein was enriched in the mitochondrial fraction (Fig 1D, lanes 1–2). Identity of the 52 kDa protein in the mitochondrial fraction was further analyzed using two different PPARγ antibodies (H100 and E8). PPARγ (H100) antibody recognizes amino acids 38–136 of PPARγ2, whereas PPARγ (E8) antibody recognizes amino acids 486–505 mapping at the C-terminus of PPARγ2 (Fig 1A). PPARγ2 expressed in HeLa cells was detected by PPARγ H100 and E8 antibodies at 57 kDa (Fig 1C, middle and bottom panels). In addition, PPARγ1 expressed in HeLa cells was detected by both PPARγ antibodies (Fig 1C, middle and bottom panels). The 52 kDa band reacted with PPARγ2 antibody in the mitochondrial fraction of BAT was recognized by PPARγ (H100) antibody but not by PPARγ (E8) antibody (Fig 1D, lane 2). Taken together, these results demonstrate that the 52 kDa protein in the mitochondrial fraction is the C-terminally truncated form of PPARγ2 (Fig 1A).

**Fig 2:**
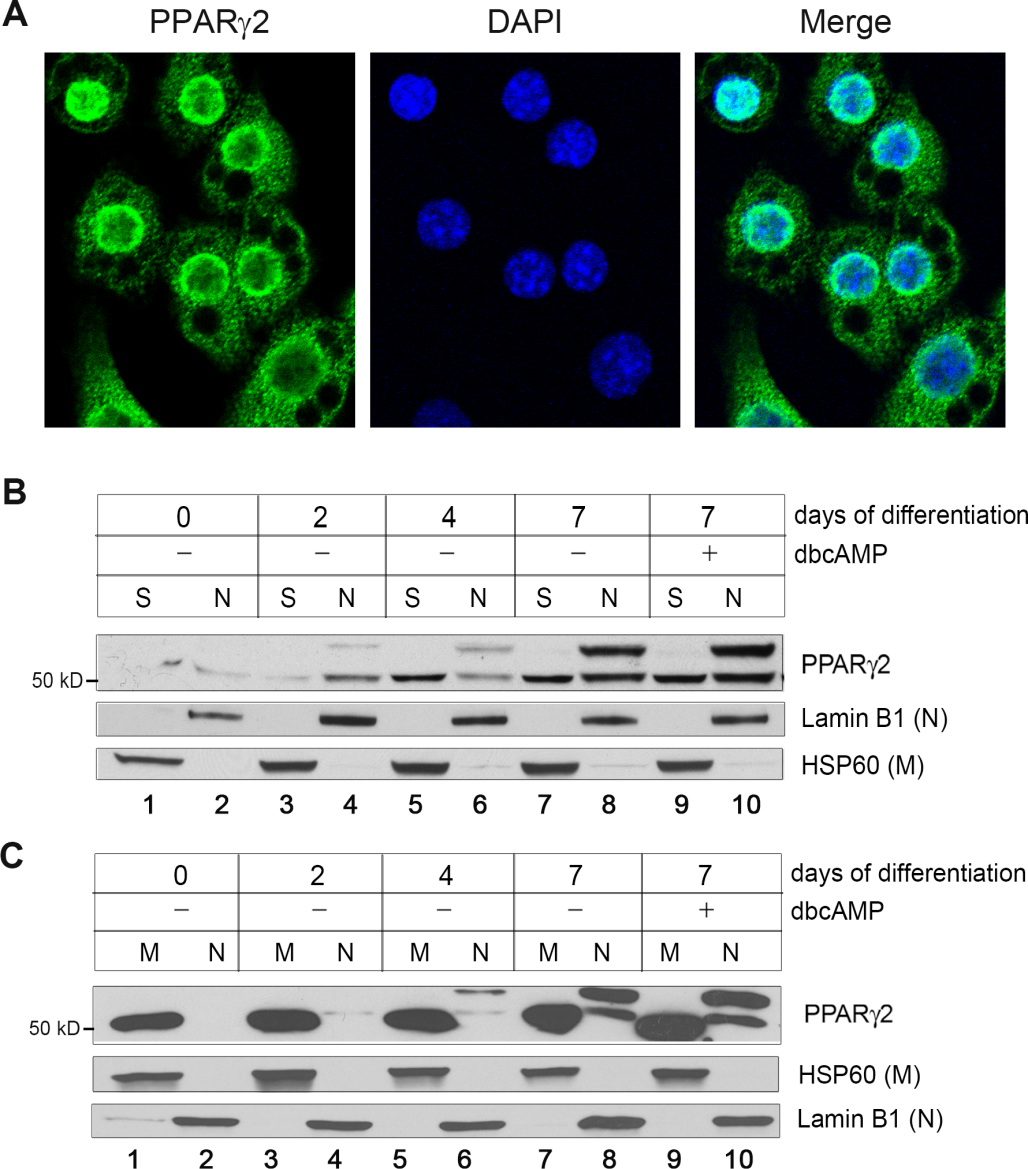
Localization of the full-length and truncated PPARγ2 during brown adipocyte differentiation. (A) Analysis of PPARγ2 localization in brown adipocytes. Brown preadipocytes were differentiated and subjected to indirect immunofluorescence using anti-PPARγ2 antibody. (B, C) Western blot analysis of the full-length and truncated PPARγ2 in subcellular fractions of brown adipocytes during differentiation. Brown preadipocytes (day 0) were differentiated for 2, 4, and 7 days, homogenized and subjected to centrifugation at 1,000 × g (N, nuclear pellets; S, nuclei-free supernatant). The nuclei-free supernatant was further centrifuged at 10,000 × g to isolate mitochondria (M).

To evaluate localization of the full-length and truncated PPARγ2 in brown adipocytes, fully differentiated brown adipocytes were subjected to indirect immunofluorescence using PPARγ2 antibody. PPARγ2 antibody-specific fluorescent signals were primarily found in the nucleus with high concentration at the nuclear envelope as well as in the cytoplasm in brown adipocytes (Fig 2A). To further determine subcellular localization of the full-length and truncated PPARγ2 during brown adipocyte differentiation, brown preadipocytes (day 0) were stimulated to undergo differentiation. The cells were collected at 2, 4 and 7 days of differentiation and subjected to nuclear and cytoplasmic fractionation. In agreement with increased PPARγ2 gene expression during brown adipocyte differentiation [12], PPARγ2 protein (57 kDa) began to become visible in the nuclear pellet at 2 days of differentiation and its levels were gradually increased during differentiation (Fig 2B). Similarly, the 52 kDa protein levels were elevated during brown adipocyte differentiation. While the full-length PPARγ2 was predominantly accumulated in the nuclear pellet (N), the truncated PPARγ2 (52 kDa) was present in both the nuclear pellet (N) and nuclei-free supernatant (S) of brown adipocytes undergoing differentiation (Fig 2B). Nuclear versus cytosolic localization is not likely regulated by cAMP-dependent signaling since treatment with cAMP did not affect localization of either the full-length or truncated PPARγ2 (Fig 2B, lanes 9–10). Next, the nuclei-free supernatant fractions were further centrifuged to isolate mitochondria. The truncated form of PPARγ2 was significantly enriched in the mitochondria of brown adipocytes undergoing differentiation (Fig 2C).

### The C-terminally truncated form of PPARγ2 is localized in the mitochondrial matrix of brown adipocytes

**Fig 3:**
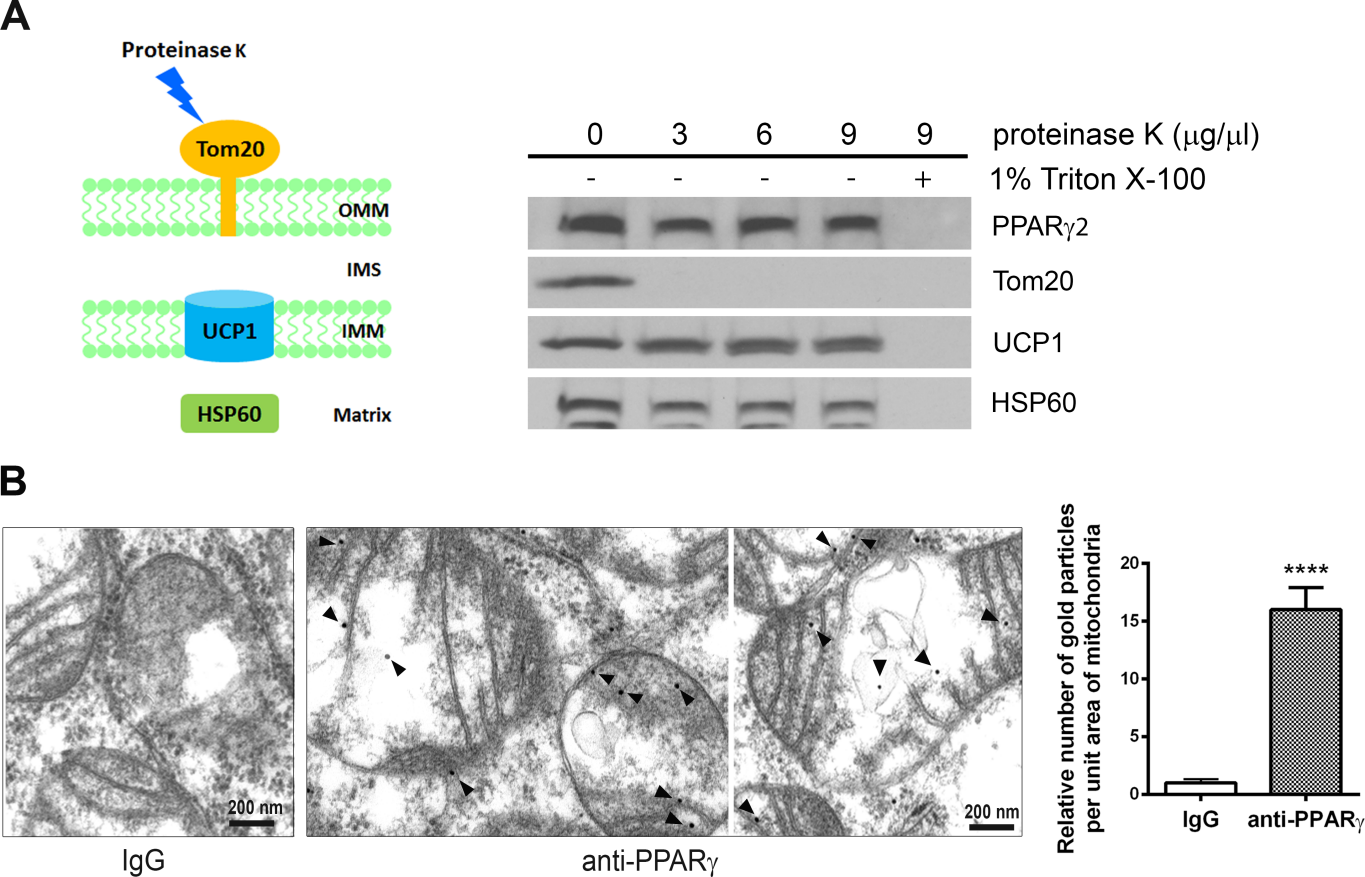
The truncated PPARγ2 localizes in the mitochondrial matrix. (A) The truncated form of PPARγ2 in mitochondria is protected from proteinase K digestion. Purified brown adipose tissue mitochondria (60 μg) were treated with increasing amounts of proteinase K in the absence or presence of 1% Triton X-100. (B) Immuno-transmission electron microscopic analysis of the truncated PPARγ2 in brown adipocytes. Black dots indicated by arrow heads represent immunogold particles reacted with PPARγ (H100) antibody. Mitochondrial localization of immunogold particles was examined in 4–5 grids per group (20–30 mitochondria/grid), and the relative number of immunogold particles localized in the mitochondria was shown in the right panel. Data are presented as the mean ± SEM. Data represent mean ±SEM. ****P<0.0001.

To further confirm that the C-terminally truncated form of PPARγ2 localizes within the mitochondria, highly purified BAT mitochondria were treated with increasing concentrations of proteinase K, which cannot penetrate the mitochondrial membranes. The outer mitochondrial membrane protein Tom20 was completely digested by proteinase K, whereas inner mitochondrial membrane protein UCP1 and mitochondrial matrix protein HSP60 were resistant to proteinase K digestion (Fig 3A). Triton X-100-mediated solubilization of mitochondria resulted in complete digestion of UCP1 and HSP60 by proteinase K. Similarly, the truncated form of PPARγ2 was protected from proteinase K digestion in the absence of Triton X-100, indicating that it does not adhere to the outer mitochondrial membrane in a nonspecific manner and is indeed localized within the mitochondria.

To determine the submitochondrial localization of the truncated form of PPARγ2, immuno-transmission electron microscopy (TEM) was carried out in fully differentiated brown adipocytes. PPARγ (H100) antibody was used because no cellular signal was detected using PPARγ2 antibody for immuno-TEM analysis. PPARγ (H100) antibody is able to detect the truncated form of PPARγ2 in mitochondria (Fig 1D). Immunogold particles corresponding to the truncated form of PPARγ2 were primarily localized in the mitochondrial matrix of brown adipocytes (Fig 3B). A small number of immunogold particles was also found closely associated with the inner mitochondrial membrane. The relative number of these immunogold particles in the brown adipocyte mitochondria was significantly higher compared with IgG control (Fig 3B).

### The C-terminally truncated form of PPARγ2 binds to the D-loop region of mitochondrial DNA

**Fig 4:**
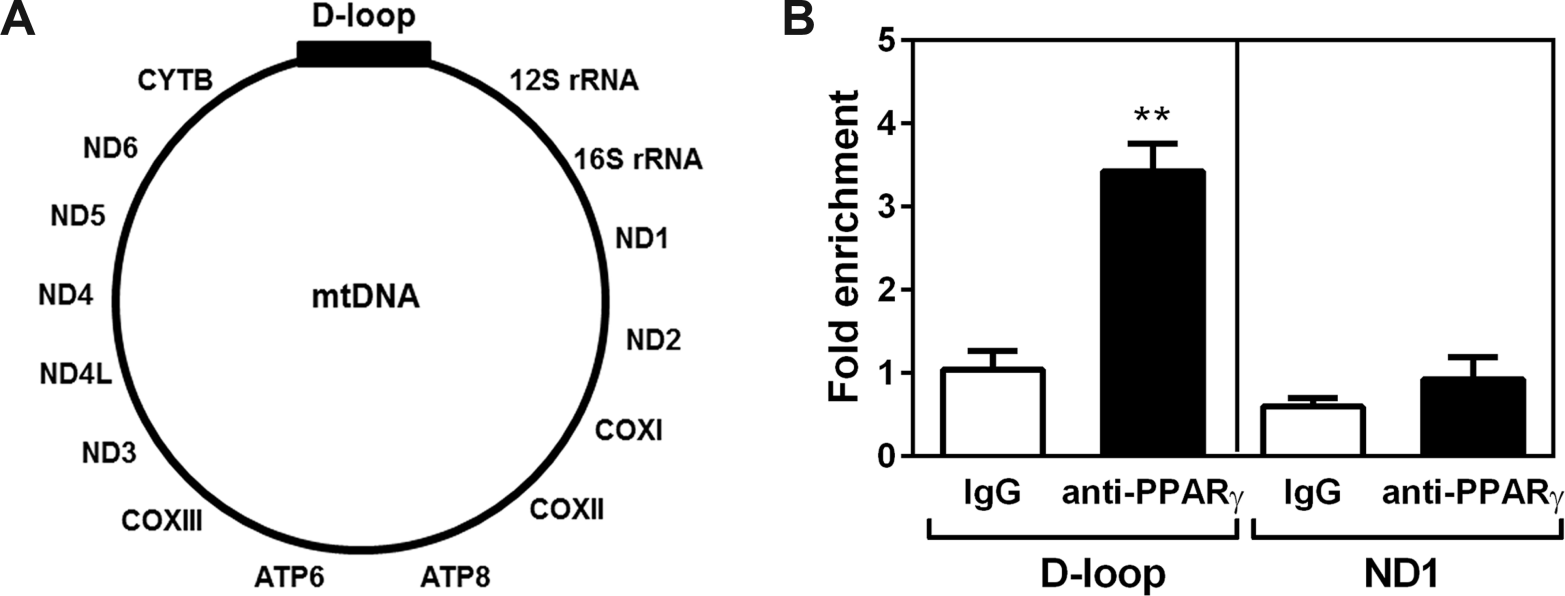
The truncated PPARγ2 binds to the D-loop region of mitochondrial DNA. (A) A schematic diagram of mitochondrial DNA (mtDNA). (B) Enrichment of the truncated PPARγ2 at the D-loop region of mtDNA in brown adipocyte mitochondria. Mitochondrial chromatin immunoprecipitation assay was carried out as described in Materials and Methods. The relative amounts of mtDNA immunoprecipitated with IgG or PPARγ (H100) antibody were analyzed by quantitative real-time PCR analysis (n = 4). Data represent mean ±SEM. **P<0.01.

The finding that the truncated form of PPARγ2 was primarily localized in the mitochondrial matrix prompted us to ask whether it regulates mitochondrial DNA transcription. Mitochondrial DNA (mtDNA) is located in the mitochondrial matrix and encodes 11 key subunits of electron transport chain (ETC) complexes I, III and IV and 2 subunits of ATP synthase (Fig 4A). The D-loop region of mtDNA contains the promoter mediating bidirectional transcription [23, 24]. To test if the truncated form of PPARγ2 is recruited to the D-loop region of mtDNA, we isolated mitochondria from brown adipocytes and carried out mitochondrial chromatin immunoprecipitation (mtChIP) assays using a ChIP grade anti-PPARγ (H100) antibody, which has been validated for its specificity and immunoprecipitation efficiency [25]. The truncated form of PPARγ2 bound to the D-loop region of mtDNA in mitochondria (Fig 4B). In contrast, no binding was detected at the coding region of ND1 gene, strengthening its specific binding to the D-loop region of mtDNA.

### Mitochondrially targeted MLS-PPARγ2 enhances mtDNA-encoded ETC gene expression and mitochondrial respiration in brown adipocytes

**Fig 5:**
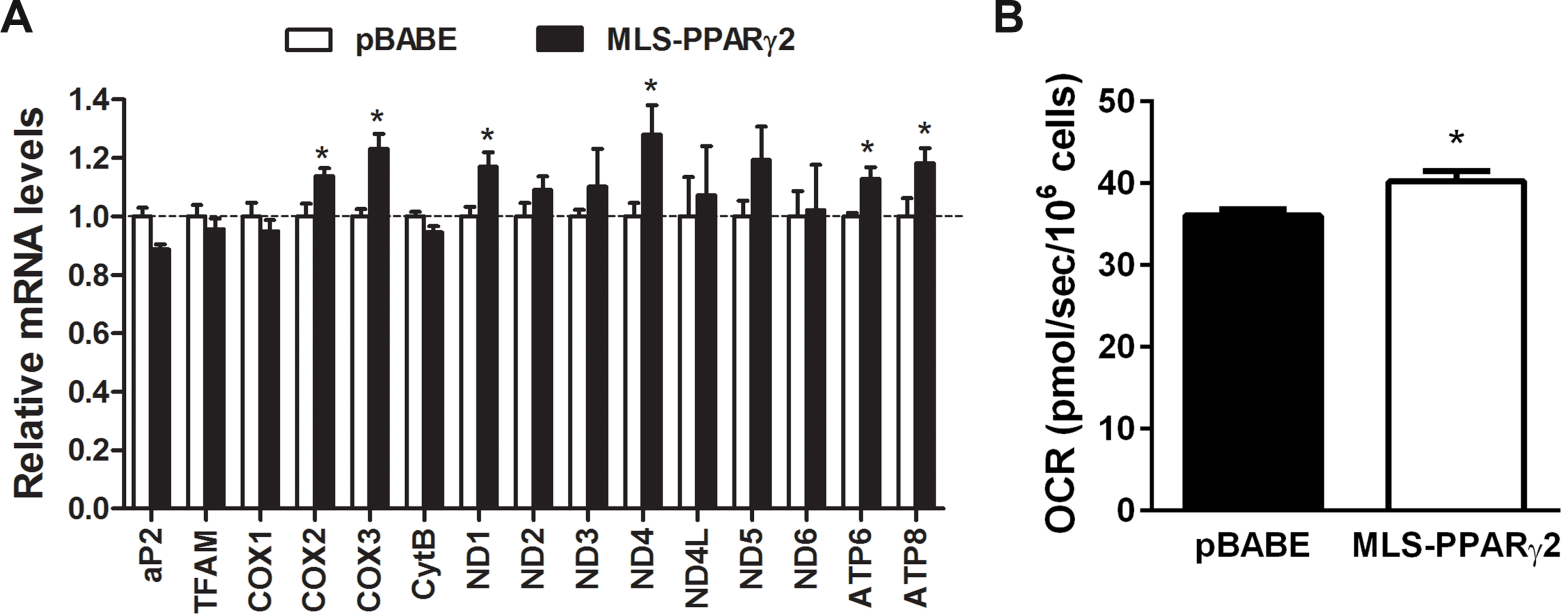
MLS-PPARγ2 increases mitochondrial respiration by modulating mtDNA-encoded ETC gene expression. (A) Expression of MLS-PPARγ2 in brown adipocytes increases mtDNA-encoded ETC gene expression. Quantitative real-time PCR was carried out in brown adipocytes expressing pBABE or MLS-PPARγ2 (n = 5). Data represent mean ±SEM. *P<0.05. (B) MLS-PPARγ2 enhances mitochondrial respiration in brown adipocytes. Cellular oxygen consumption rates (OCR) were measured at baseline and after injection of antimycin A (n = 6). The value of mitochondrial respiration was determined by subtracting antimycin A-independent non-mitochondrial respiration. Data represent mean ±SEM. *P<0.05.

Given that the truncated form of PPARγ2 bound to the D-loop region of mtDNA, we wanted to test if this protein regulates mtDNA-encoded ETC gene expression in mitochondria. We constructed MLS-PPARγ2 that contains a mitochondrial matrix localizing sequence (MLS) fused to the N-terminus of the protein. In frame insertion of the MLS directs the protein to the mitochondria and thus has been used to assess the function of nuclear transcription factors specifically in the mitochondria without its effect on gene expression in the nucleus [19, 26–28]. MLS-PPARγ2 was stably expressed in brown preadipocytes by retrovirus-mediated gene transfer. A previous study has shown that PPARγ2 expression in fibroblasts stimulates adipogenic differentiation [29]. We also observed that PPARγ2 expression in brown preadipocytes partially induced adipocyte differentiation without a differentiation cocktail, whereas no adipocyte differentiation was stimulated by MLS-PPARγ2 expression in brown preadipocytes (data not shown). This indicates that MLS-PPARγ2 has no effect on gene expression in the nucleus. Further supporting this, expression levels of aP2, which is a PPARγ target gene and an adipogenic marker, were not different between MLS-PPARγ2- and empty vector-expressing brown adipocytes (Fig 5A). Mitochondrial transcription factor A (TFAM) is a key regulator of mtDNA transcription [24]. TFAM is encoded by nuclear genome and imported to mitochondria. Gene expression analysis showed that TFAM was comparably expressed in MLS-PPARγ2- and empty vector-expressing brown adipocytes. Despite the same levels of TFAM, expression of several mtDNA-encoded ETC genes was elevated in MLS-PPARγ2-expressing brown adipocytes compared to control brown adipocytes (Fig 5A), suggesting a direct effect of MLS-PPARγ2 on mtDNA-encoded ETC gene expression. Next, to determine the effect of increased ETC gene expression on mitochondrial respiration, cellular oxygen consumption rates (OCR) were measured at baseline and after injection of a mitochondrial electron transport inhibitor, antimycin A. Mitochondria-dependent respiration was determined by subtracting antimycin A-independent non-mitochondrial respiration. In agreement with an increase in mtDNA-encoded ETC gene expression by MLS-PPARγ2, mitochondrial respiration was slightly enhanced in MLS-PPARγ2-expressing brown adipocytes compared to control brown adipocytes (Fig 5B). Taken together, these results indicate that mitochondrial PPARγ2 plays a role in mitochondrial respiration via regulating mtDNA-encoded ETC gene expression in brown adipocytes.

## Discussion

The present study identifies the truncated form of PPARγ2 (52 kDa) that is highly enriched in brown adipocyte mitochondria and regulates mtDNA-encoded ETC gene expression. While the full-length PPARγ2 was predominantly localized in the nucleus, the truncated form of PPARγ2 was located in the nucleus, cytoplasm, and mitochondria of brown adipocytes. Cytoplasmic localization of this truncated protein is not surprising because nuclear-cytoplasmic shuttling of PPARγ1 and PPARγ2 has been reported. PPARγ1 and PPARγ2 contain nuclear import and export signals at the DNA-binding domain (DBD) and the ligand-binding domain (LBD), respectively [30]. Accordingly, their nuclear-cytoplasmic shuttling is mediated by a nuclear importer importin α/β and a nuclear exporter CRM1 [30]. The truncated PPARγ2 lacks the C-terminal end of PPARγ2 (at least amino acids 486–505) that in part consists of the activation function 2 (AF2) domain (amino acids 436–505). The AF2 domain is required for ligand-dependent activation of PPARγ2 via interaction with coactivators and subsequent degradation via the ubiquitin proteasome system [31, 32]. It is not likely that lack of the C-terminal end of PPARγ2 increases cytoplasmic translocation since the AF2 domain does not regulate nuclear-cytoplasmic shuttling of PPARγ1 [33]. Rather, it is more probable that the C-terminal end of PPARγ2 retains the full-length PPARγ2 better in the nucleus via interaction with nuclear coactivators. How does the truncated form of PPARγ2 translocate to mitochondria? There are growing number of evidence that nuclear transcription factors translocate to mitochondria despite the absence of a mitochondrial localization sequence. Several studies have shown that mitochondrial heat shock protein 70 (mtHSP70) and voltage-dependent anion channel (VDAC) serve as a transport route for proteins that lack a mitochondrial localization sequence [2, 3, 34, 35]. Thus it would be interesting to examine whether mtHSP70 and/or VDAC assist mitochondrial import of the truncated form of PPARγ2.

Several studies have reported mitochondrial translocation of truncated nuclear receptors such as thyroid receptor (TRα1 p43), progesterone receptor (PR-M), and retinoid X receptor (RXRα p44) in a tissue- or cell-specific manner [36–38]. These truncated proteins are produced by multiple pathways including translation at the internal initiation site, alternative splicing, and enzymatic cleavage. Casas et al. previously reported that a 45 kDa protein related to PPARγ2 is present in the mitochondria of brown adipose tissue [16]. It’s not clear whether this protein is identical to the truncated PPARγ2 we found in the present study. However, both findings demonstrate that the truncated forms of PPARγ2 are predominantly located in brown adipose tissue mitochondria. Cytoplasmic PPARγ2 has been shown in 3T3-L1 adipocytes, but its truncated form has not been reported [39]. We did not detect the truncated form of PPARγ2 at 52 kDa in subcutaneous and visceral adipose tissue (data not shown). This may suggest that the truncated form of PPARγ2 is specifically produced in brown adipocytes. The mechanism by which the truncated form of PPARγ2 originates in brown adipocytes remains to be determined.

Collectively, our data suggest that mitochondrial PPARγ2 affects mitochondrial respiration through influencing the expression of mtDNA-encoded ETC genes in brown adipocytes. Many ETC subunits encoded by nuclear DNA assemble as subcomplexes in the mitochondrial matrix and their redistribution to the inner mitochondrial membrane are accelerated by mtDNA-encoded ETC subunits [40, 41]. Thus the mechanism by which mitochondrial PPARγ2 regulates mtDNA-encoded ETC gene expression may provide an additional level of control for efficient ETC complex formation in the inner mitochondrial membrane of brown adipocytes. Enhanced mitochondrial respiration through the ETC complexes is critical for UCP1-mediated heat production in brown adipocytes.
